# Spitting Blood

**Published:** 1882-04

**Authors:** 


### SPITTING BLOOD.

FROM AN OLD PATIENT TO A CLERGYMAN WHO HAD HEMORRHAGE.

"FIVE years ago, after having had a bad spasmodic cough for about three months, I suddenly bled about a gill, cough continuing and slight bleeding for two months more; was feeble all summer and following winter; tried Dr. —— and then Dr. —— and S——, who, I thought, helped me much, and in great joy I wrote him a letter, which he published. But I think my recovery after all was owing to out-door exercise, regularly and perseveringly kept up. I called on Dr. ——, a year ago last spring, when I considered myself pretty well. He examined my lungs and told me they were then sound and working well, that my recovery was complete. Last winter, however, I had no time for any thing, and by hard work and confinement brought on my former difficulty, cough; bleeding in April of last year about as before, repeated two or three times, the last the fifth of July. Did not preach from June to October. I consulted no physician during this sickness, relying on my former experience and my former method—out-door exercise. But Dr. Hall's advice was what I followed, taken when I before consulted him for such difficulties.

"First. All the gentle out-door exercise possible, short of fatigue. Never get exhausted.

"Second. All the good plain food you can digest; avoid pastries and cakes. The best diet is plain bread and beef.

"Third. Try and have pure air always. Connected with this advice of Dr. ——, I used, by the advice of a Boston physician, a tablespoon of Bourbon whisky three times a day before eating. Furthermore, I rubbed my chest daily with a crash towel, then a strong liniment. I think a warm bath in a warm room once a week all-sufficient in the matter of bathing. I think the great thing is out-door exercise—that is the cure. It was very obstinate—so was I. I persevered, almost despairing many times; but the last Sabbath in October I tried preaching. For four Sundays it was hard work. From that time until the present, however, my voice never was clearer and stronger. I have preached and talked just as much as I chose. Henceforth I hope not to over-do, and to have a plenty of out-door exercise. I would advise you by all means to go to Dr. ——. I think he is the man of all others. Be of good courage. I do think that no one need die of consumption; as long as one can walk a quarter of a mile he can be cured.

"In your case all violent or protracted exercise ought for a time to be avoided.

"P S.—Coarse brown or Graham bread is better than all wheat bread. A free passage daily of the bowels is of vital importance. If brown bread sours on your stomach, do not eat it. The idea is to get up a good digestion, and so build up the general strength."

The above letter incorporates our own sentiments, and it may be suggestive to state that the person to whom it was addressed declined placing himself under our care because we refused to prescribe any medicine, showing, as far as it goes, that druggery is the folly of the age, not from the prescription of the physician, but from the folly of the people, added to their impatience; they are willing to do any thing that will make them feel better for the moment rather than adopt means, which, although slower in the promotion of beneficial results are radical, safe, and lasting, any one of which three things are of the first importance. A wise man ought to want to be safely dealt with, to be thoroughly cured, and to be cured lastingly, yet such are the views practically of very few people, and every educated physician has cause to lament it constantly.

